# Primary osteosarcoma of the urinary bladder treated with external radiotherapy in a patient with a history of transitional cell carcinoma: a case report

**DOI:** 10.1186/1752-1947-4-70

**Published:** 2010-02-24

**Authors:** Christos Papandreou, Antigoni Skopelitou, George Kappes, Housam Daouaher

**Affiliations:** 1 Department of Urology, General Hospital of Arta, Greece; 2 Department of Pathology, General Hospital of Arta, Greece; 3 Department of Radiology, General Hospital of Arta, Greece

## Abstract

**Introduction:**

Primary osteosarcoma is one of the rare tumors affecting the urinary bladder. The occurrence of osteosarcoma in a patient with a long history of transitional cell carcinoma of the bladder is even more uncommon.

**Case presentation:**

We present the case of a 74-year-old Greek man who was diagnosed with osteosarcoma 10 years after he had been diagnosed with transitional cell carcinoma of the bladder from which he had been free from recurrences for the past three years. Our patient was treated for the osteosarcoma with transurethral resection of bladder tumor and external beam radiation therapy. He died eight months after the diagnosis, suffering poor quality of life in the last months.

**Conclusion:**

Osteosarcoma of the bladder has a dismal prognosis. External beam radiation therapy as an adjunct to transurethral resection of bladder tumor not only provides no benefit to patients with primary osteosarcoma of urinary bladder, but also may be associated with poor quality of life.

## Introduction

Osteosarcoma of the urinary bladder is a rare tumor. To date, only 32 cases have been reported using several treatment modalities [1-3]. We present another case of this neoplasm, which was managed with transurethral resection and external beam radiation therapy.

## Case presentation

A 74-year-old Greek man presented in June 2008 with gross hematuria. Our patient had been previously treated for non-muscle invasive pTa, G2 transitional cell carcinoma of the bladder (TCC). The first transurethral resection of bladder tumour (TURBT) had been performed ten years before, while the last was performed three years before. The last follow up cystoscopy had been performed twelve months before. Our patient was asymptomatic thereafter, until June 2008.

**Figure 1:**
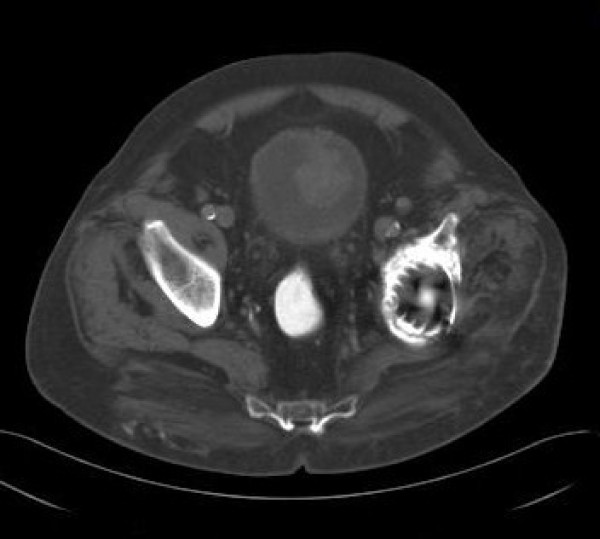
**Computed tomography scan shows a large solitary polypoid tumour arising from the bladder dome**. Also notice left hip arthroplasty.

Then, at cystoscopy, a large solitary polypoid, yellow-white tumor was found on the superior surface of the bladder. A computed tomography (CT) scan revealed a heterogeneous mass arising from the bladder dome with evidence of invasion of the bladder wall (figure 1). There was no evidence of pelvic or abdominal lymphadenopathy. Subsequent transurethral resection was extremely difficult and incomplete because of hardness of the tissue. Pathological examination of retrieved specimen revealed a highly pleomorphic and hypercellular neoplasm composed of spindled sarcomatous cells with characteristic formation of malignant osteoid, as well as an abundant chondroid matrix.

**Figure 2:**
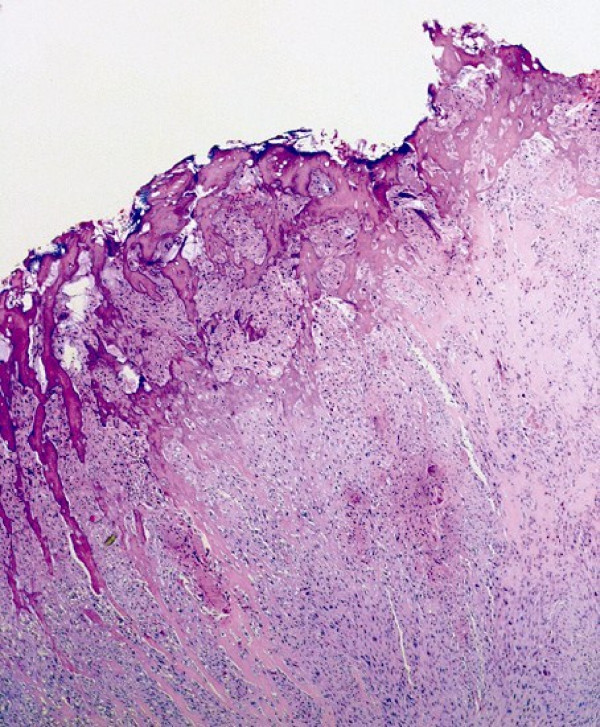
**Cancellous bone and malignant, lace-like osteoid formation by the spindled sarcomatous cells and in between these cells chondroid matrix (HE, × 100)**.

**Figure 3:**
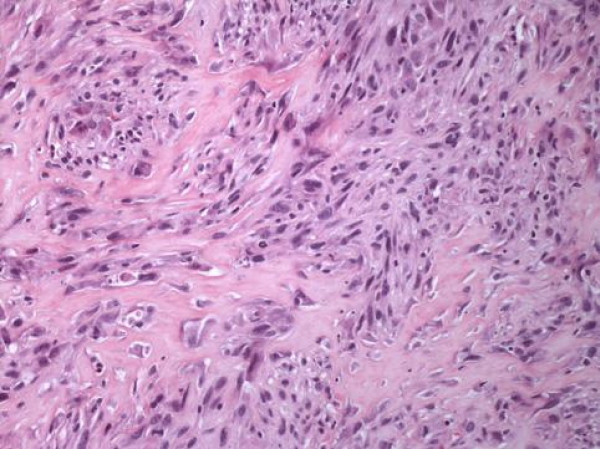
**Malignant, lace-like osteoid formation**. Notice the nuclear polymorphism of the malignant cells producing malignant woven bone. (HE, × 200)

Immunohistochemical analysis performed on paraffin-embedded tissue sections was negative for pan-cytokeratin 7 and 20, epithelial membrane antigen (EMA), as well as a smooth muscle actin, desmin, CD34 and CD68. Vimentin and p53 were strongly expressed in more than 95% of the neoplasmatic cells. No transitional cell carcinoma or carcinosarcoma elements were evident (figures 2 and 3). These findings supported the diagnosis of primary osteogenic sarcoma of the urinary bladder. Other localization studies including a chest X-ray and a bone scan were unremarkable.

**Figure 4:**
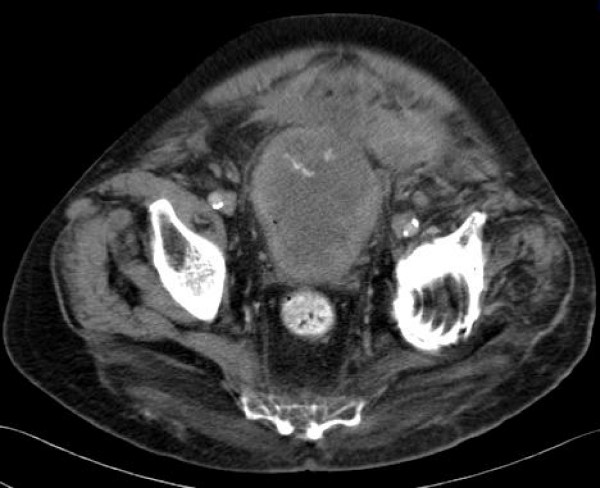
**Computed tomography scan seven months afterdiagnosis shows extravesical tumour extension and bowel invasion**.

Due to the significant comorbidity of our patient, who had a coronary artery disease and restrictive respiratory disease, he was offered partial cystectomy. However, he refused this option and underwent external beam radiation therapy (EBRT) instead. Our patient declined to receive adjunctive chemotherapy. In the following months, he had two episodes of proven urinary tract infection. Seven months after diagnosis, he developed ascites. A CT scan at that point showed extravesical tumor extension and bowel infiltration (figure 4). Our patient died eight months after diagnosis, experiencing severe lower urinary tract symptoms (LUTS) and abdominal pain the last months of his life.

## Discussion

Only 32 cases of primary osteosarcoma of the bladder have been so far reported in the English literature [1-3]. To the best of our knowledge, this is the fourth reported case where this type of tumor occurs in a patient with a history of TCC or simultaneously with TCC [4-6].

Osteosarcoma should be distinguished from other bone-forming tumors, such as carcinosarcoma and transitional cell carcinoma with osseous metaplasia. It is not always easy to distinguish extraskeletal osteosarcoma from other sarcomas, which rarely produce metaplastic bone such as malignant fibrous histiocytoma, synovial sarcoma and epithelioid sarcoma. In most of these neoplasms, osteoid or bone formation is confined in a small portion of the tumor and is relatively well differentiated, without the disorderly pattern and cellular/nuclear polymorphism/pleomorphism of osteosarcoma. The characteristic microscopic pattern of our tumor, that is, the malignant, lace-like osteoid, produced in large amounts by the spindled sarcomatous cells, and the presence of chondroid matrix between these cells along with the above-mentioned immunoprofile and the absence of concurrent epithelial malignancy, supported the diagnosis of primary osteosarcoma of urinary bladder [7-9].

The prognosis of bladder osteosarcoma is very poor, with most patients dying from the disease within six months. The tumor tends to be locally aggressive. Distant metastases are uncommon and are usually confined to the lung. Recommendations to improve survival have included radical cystectomy with radiotherapy and/or chemotherapy, but most patients die of this disease regardless of the type of treatment [1,10].

However, there are publications that report successful treatment with a long follow- up. One patient was free of such disease 36 months after partial cystectomy [10] and another survived with no evidence of recurrence after 51 months of combination treatment with radical cystectomy and chemotherapy [11]. A case of bladder osteosarcoma with markedly remission of pulmonary metastases after chemotherapy has also been reported [1].

In our case, our patient had a long history of TCC and also had significant comorbidity. He was thoroughly informed about the disease and finally decided to undergo EBRT. Although he lived few months more than usual, during the last four months, he suffered severe LUTS, abdominal pain and was heavily medicated with opioid analgesics. For the above-mentioned symptoms, there was no obvious etiology other than radiotherapy.

## Conclusion

Osteosarcoma of the bladder is a rare tumor with a dismal prognosis. External beam radiation therapy as adjunct to transurethral resection seems to provide no benefit for patients with primary osteosarcoma of urinary bladder. Moreover, it may be associated with poor quality of life and thus should be better to be avoided.

## Abbreviations

CT: Computed tomography; TCC: transitional cell carcinoma; EBRT: external beam radiation therapy; TURBT: transurethral resection of bladder tumor; LUTS: lower urinary tract symptoms.

## Consent

Written informed consent was obtained from our patient for publication of this case report and accompanying images. A copy of the written consent is available for review by the Editor-in-Chief of this journal.

## Competing interests

The authors declare that they have no competing interests.

## Authors' contributions

CP was involved in patient care, review of literature and writing of the manuscript. AS performed the histological examination and reviewed the literature. GK was involved in radiological evaluation of our patient. HD was involved in patient care and writing supervision. All authors read and approved the final manuscript.
